# What is the prognostic impact of FDG PET in locally advanced head and neck squamous cell carcinoma treated with concomitant chemo-radiotherapy? A systematic review and meta-analysis

**DOI:** 10.1007/s00259-018-4065-5

**Published:** 2018-06-09

**Authors:** Pierluigi Bonomo, A. Merlotti, E. Olmetto, A. Bianchi, I. Desideri, A. Bacigalupo, P. Franco, C. Franzese, E. Orlandi, L. Livi, S. Caini

**Affiliations:** 1 Radiation Oncology, Azienda Ospedaliero – Universitaria Careggi, University of Florence, largo Brambilla 3, 50134 Florence, Italy; 2 Radiation Oncology, Azienda Ospedaliera S.Croce e Carle, Cuneo, Italy; 3 Nuclear Medicine Department, Azienda Ospedaliera S.Croce e Carle, Cuneo, Italy; 4 Radiation Oncology Department, Ospedale Policlinico San Martino, Genoa, Italy; 5 Department of Oncology, Radiation Oncology, University of Turin, Turin, Italy; 6 Department of Radiotherapy and Radiosurgery, Humanitas Cancer Center and Research Hospital, Rozzano, Italy; 7 Radiotherapy 2 Unit, Fondazione IRCCS Istituto Nazionale dei Tumori, Milan, Italy; 8 Cancer Risk Factors and Lifestyle Epidemiology Unit, Cancer Research and Prevention Institute (ISPO), Florence, Italy

**Keywords:** Head and neck cancer, Radiotherapy, ^18^F-Fluorodeoxyglucose (FDG) positron emission tomography, Metabolic tumour volume

## Abstract

**Purpose:**

Evidence is conflicting on the prognostic value of ^18^F-fluorodeoxyglucose (FDG) positron emission tomography (PET) in head and neck squamous cell carcinoma. The aim of our study was to determine the impact of semiquantitative and qualitative metabolic parameters on the outcome in patients managed with standard treatment for locally advanced disease.

**Methods:**

A systematic review of the literature was conducted. A meta-analysis was performed of studies providing estimates of relative risk (RR) for the association between semiquantitative metabolic parameters and efficacy outcome measures.

**Results:**

The analysis included 25 studies, for a total of 2,223 subjects. The most frequent primary tumour site was the oropharynx (1,150/2,223 patients, 51.7%). According to the available data, the majority of patients had stage III/IV disease (1,709/1,799, 94.9%; no information available in four studies) and were treated with standard concurrent chemoradiotherapy (1,562/2,009 patients, 77.7%; only one study without available information). A total of 11, 8 and 4 independent studies provided RR estimates for the association between baseline FDG PET metrics and overall survival (OS), progression-free survival (PFS) and locoregional control (LRC), respectively. High pretreatment metabolic tumour volume (MTV) was significantly associated with a worse OS (summary RR 1.86, 95% CI 1.08–3.21), PFS (summary RR 1.81, 95% CI 1.14–2.89) and LRC (summary RR 3.49, 95% CI 1.65–7.35). Given the large heterogeneity (*I*^2^ > 50%) affecting the summary measures, no cumulative threshold for an unfavourable prognosis could be defined. No statistically significant association was found between SUV_max_ and any of the outcome measures.

**Conclusion:**

FDG PET has prognostic relevance in the context of locally advanced head and neck squamous cell carcinoma. Pretreatment MTV is the only metabolic variable with a significant impact on patient outcome. Because of the heterogeneity and the lack of standardized methodology, no definitive conclusions on optimal cut-off values can be drawn.

**Electronic supplementary material:**

The online version of this article (10.1007/s00259-018-4065-5) contains supplementary material, which is available to authorized users.

## Introduction

Head and neck cancer is the sixth most common malignant tumour, with increasing incidence worldwide [1]. In over 95% of cases, the disease arises from the epithelial layer of the mucosa lining the upper aerodigestive tract. Due to the absence of anatomical barriers, the abundant lymphatic drainage of the neck and the usually infiltrative pattern of growth of head and neck squamous cell carcinoma (HNSCC), in about 60% of patients the diagnosis is made at an advanced locoregional stage. In order to maximize the likelihood of disease cure, multimodality treatment is usually needed. Therapeutic management is often challenging: both primary radical surgery and concurrent chemoradiotherapy are burdened with a high rate of posttreatment complications, acute and long-term toxicities [2] and a marked detrimental effect on quality of life. Notwithstanding the refinement of treatment strategies that has taken place in last 20 years, the prognosis of HNSCC remains severe, with a cumulative 5-year overall survival (OS) rate of 45–55% [3] in patients with locally advanced disease. The prevalent pattern of failure in the overall population is locoregional: about 50% of first events of relapse occur at the primary tumour site and/or in the neck, in the vast majority (about 90%) within the first 2 years after treatment.

Taking into account that the patient’s outlook can be substantially influenced by clinical factors with large variability existing among the different subsites of disease, a series of common features contribute to the severe prognosis of locally advanced HNSCC; these include the suboptimal efficacy of the standard “one size fits all” multimodal approach, the large proportion of frail patients who are noncompliant with intensive therapy, and the absence of biomarkers. In this regard, the only notable exception is the human papillomavirus (HPV). In the last 15 years, a major epidemiological shift has taken place in western countries due to the rising incidence of HPV-associated oropharyngeal cancer [4], reducing the dominance of the classical phenotype of HNSCC resulting from alcohol and tobacco-induced field cancerization. A positive HPV status was recognized as an independent favourable prognostic factor in a series of correlative prospective studies and in an unplanned secondary analysis of the randomized phase 3 RTOG 0129 trial [5]. Overall, HPV positivity is associated with a reduction in the risk of death and disease progression of about 60%.

Although major progress has been achieved in unravelling key molecular pathways involved in HNSCC pathogenesis [6], at present no biomarkers are available in clinical practice apart from HPV status. Prognostic information is therefore critically lacking in the management of patients affected by HNSCC. Next to individual genomic profiling, an alternative strategy which has been explored in recent years is to integrate molecular imaging into precision oncology care, exploiting the potential of imaging as a biomarker. The possibility of linking the information obtained from medical images with personalized treatment forms the core of “theragnostics”, an term that has been used particularly in the context of radiation therapy [7]. In a hallmark review published in 2000, Ling et al. [8] suggested that the evolution of molecular imaging could facilitate the development of customized dose delivery in the era of intensity-modulated radiotherapy (IMRT). As foreseen by Ling and colleagues, in the last 15 years molecular imaging has been increasingly implemented in the management of HNSCC, in particular ^18^F-fluorodeoxyglucose (FDG) positron emission tomography (PET). The fundamental prerequisite is the ability to image physiopathological processes occurring within a tumour or its microenvironment. The use of FDG allows the characterization of the metabolic activity of a defined tumour burden. In HNSCC, available evidence supports the role of FDG PET in primary target definition for radiotherapy planning [9], staging [10] and posttreatment response assessment [11]. However, its potential impact on patient outcomes is an unresolved issue. The aim of this work was to define the relevance of semiquantitative and qualitative FDG PET features as prognostic biomarkers in the curative setting of locally advanced head and neck cancer.

## Materials and methods

In accordance with the PRISMA (Preferred Reporting Items for Systematic Reviews and Meta-Analyses) statement [12], a systematic review of the literature was conducted. Relevant articles were identified in two databases (MEDLINE and Embase) over a 10-year period (1 January 2007 to 28 February 2017) using the appropriate terminology as described in Appendix 1 of the Supplementary material. Conference proceedings of main international conferences (ASCO, ASTRO, ESMO, ESTRO, ECCO) were also searched. The reference lists of the articles reviewed as full texts were also searched manually. The literature search strategy was based on the PICO methodology [13], as discussed in the following sections.

### Population

The target population of our analysis consisted of adult patients (>18 years of age) treated with curatively intended radiotherapy, concurrent chemoradiotherapy or radiotherapy combined with targeted therapy for locally advanced HNSCC. Primary surgery and induction chemotherapy were not allowed. In view of the known heterogeneity among different head and neck subsites, we sought to assess whether the impact of metabolic parameters could be observed in specific disease entities or in HNSCC taken as a whole. In addition, information on the radiotherapy technique used and the schedule of systemic therapy administered was collected when available. To provide evidence-based support for the analysis, the published literature was categorized according to the type of study design: all case series except those with fewer than 20 patients, literature reviews and consensus statements were eligible. Only studies in the English language were included.

### Interventions

Upon inclusion in the analysis, adequate information on FDG PET metrics (semiquantitative parameters and/or qualitative scores) had to be retrieved from the studies analysed. Studies focusing on tracers other than FDG and on integrated PET/MRI were excluded. Since the main aim of this review was to investigate the potential impact of specific metabolic data on HNSCC prognosis, the following parameters were considered as main interventions: standardized uptake values (SUV_max_, SUV_mean_, SUV_peak_), metabolic tumour volume (MTV) and total lesion glycolysis (TLG). These parameters were defined according to reference guidelines [14], as follows:

- SUV (body-weighted): the concentration of FDG in a given region of interest (ROI) or volume of interest (VOI; expressed in kilobecquerels per millilitre) divided by the ratio between administered activity (corrected for radioactive decay at the time of scanning) and the body weight of the patient
- SUV_max_: the highest SUV of pixels (or voxels) in a given ROI (or VOI)
- SUV_mean_: the mean SUV of pixels (or voxels) in a given ROI (or VOI)
- SUV_peak_: SUV_mean_ within a 1-cm^3^ spherical VOI centred on the voxels with the highest uptake
- MTV: the VOI segmented using a fixed threshold (usually 41% or 50%) of FDG-avid lesions
- TLG: the product of the VOI average SUV (SUV_mean_) and the corresponding MTV

Standardized qualitative interpretations of FDG PET scans were also considered interventions, if rigorously defined. In addition, the included studies were further analysed according to the timing of the FDG PET scans, whether performed before, during or after treatment.

### Comparators

When available, different clinical factors other than the metabolic FDG PET parameters discussed above were defined as “comparators” if analysed as potential prognostic biomarkers.

### Outcomes

Ultimately, we sought to assess whether intrinsic features on FDG PET retain prognostic significance in terms of outcome. Therefore, we searched for a potential correlation between the interventions (as described above) and locoregional control (LRC), progression-free survival (PFS) and OS at a minimum follow-up of 1 year. These outcome measures were defined as follows:

- LRC: the time from randomization (or study initiation) to local and/or regional disease progression
- PFS: the time from randomization (or study initiation) to disease progression or death
- OS: the time from randomization (or study initiation) to death from any cause

Studies in which the main outcome measure was not consistent with the definition of the prespecified efficacy endpoints were excluded. Studies performed to assess the diagnostic accuracy of FDG PET as well as “in-silico” radiotherapy planning analyses were also excluded.

### Statistical analysis

Baseline demographics, patient and disease characteristics, treatment features and outcome data were collected by three authors (P.B., A.M., E.O.), verified by two reviewers (I.D., S.C.) and summarized using descriptive statistics. From all studies included in the literature review, we extracted the most adjusted estimate of relative risk (RR), including odds ratio and hazard ratio (HR), for the association between each of the metabolic parameters (SUV_max_, SUV_mean_, SUV_peak_, MTV and TLG) and each of the patient outcomes (OS, PFS and LRC). When there were two or more independent RR estimates, these were transformed into logRR and the corresponding variance using the formula of Greenland [15] and pooled using random effects models to obtain a summary RR (SRR) and corresponding 95% confidence intervals (CI). We assessed the heterogeneity between studies using the *I*^2^ statistic, which is interpreted as the percentage of the variability that is attributable to true heterogeneity rather than chance. Larger values of *I*^2^ denote greater between-estimate heterogeneity; values of *I*^2^ below 50% are considered acceptable. We did not perform subgroup analysis and meta-regression because of the limited sample size. Finally, we evaluated the presence of publication bias using the funnel plot of Begg and Mazumdar [16] and the regression test of Egger et al. [17]. The meta-analysis was conducted using the *metan* command in Stata version 14 (Stata Corp, College Station, TX).

## Results

### Data collection and analysis

**Fig. 1:**
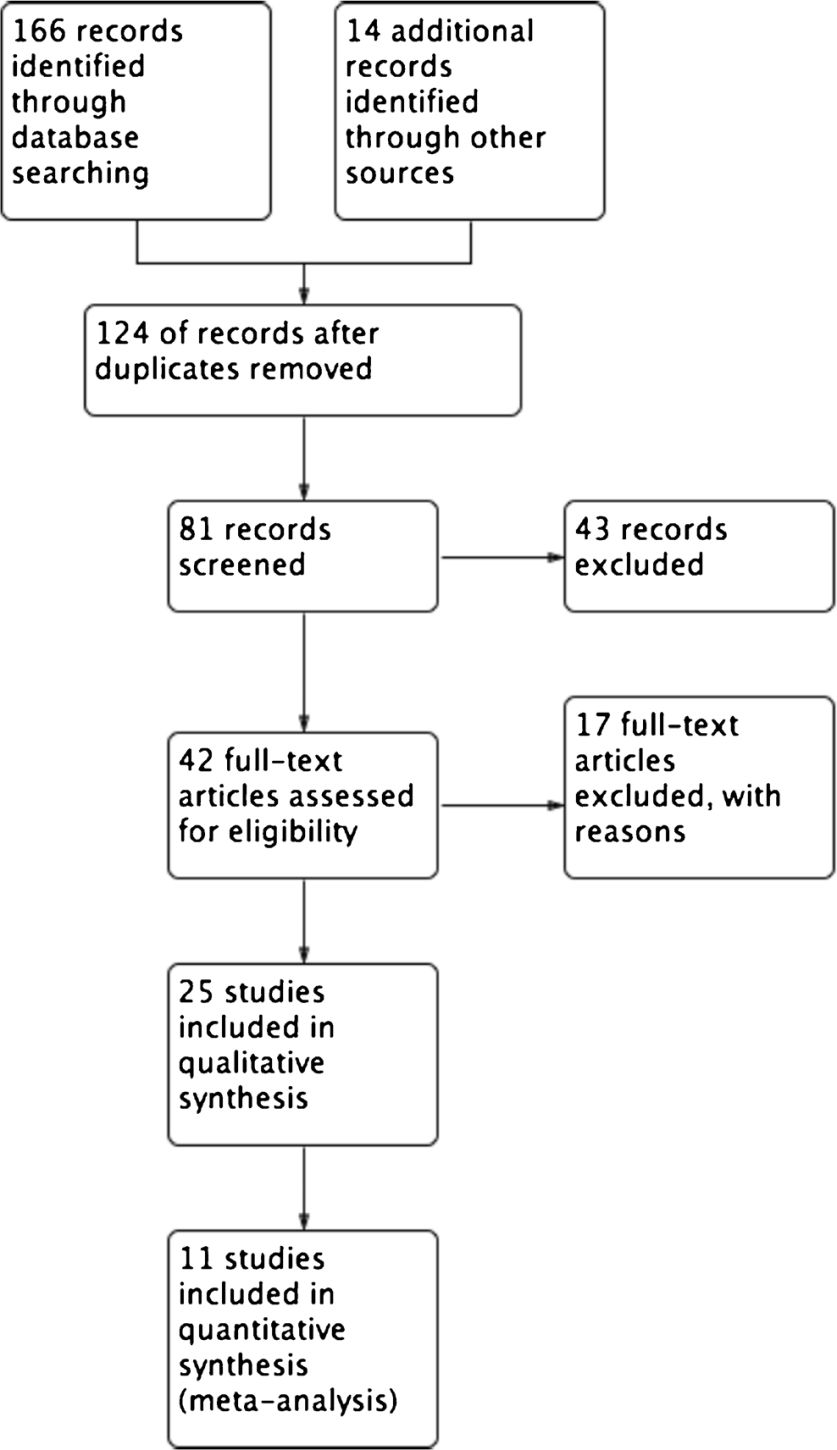
Flow chart of the literature search

Two authors (P.B., A.M.) independently examined the titles and abstracts of each search record, and retrieved the full text articles for potentially eligible studies. The full texts were further examined according to the inclusion criteria. Discrepancies were resolved by consensus. Data were extracted by the two authors using a data collection form. Overall, of 180 studies identified using the predefined search criteria, 81 were screened by assessment of the abstracts (Fig. 1). Of these screened studies, 42 were evaluated for eligibility, and 25 [18–44] satisfied the inclusion criteria and were therefore analysed fully. The whole reference lists of the eligible studies and the reasons for exclusion are available in Appendix 2 of the Supplementary material. In terms of study design, most included studies (21/25, 84%) were retrospective. Two papers were initially retrieved in abstract form [33, 41] and updated as soon as the full versions became available [34, 42]. One study [29] had limited data on the disease and treatment characteristics collected in most patients, but provided adequate information on FDG PET variables and outcomes.

### Patient characteristics

The overall population consisted of 2,223 patients (Table 1). The median age of the whole cohort was 59 years (range 48–68 years). Most patients (1,875/2,223, 84.3%) were men. Only 3 of the 25 studies [31, 32, 40] provided information about tobacco exposure in terms of pack-years. Generic information on subjects with a smoking history was reported in six additional studies [22, 28–30, 39, 43] (81.4%, 82.6%, 67.3%, 80%, 90.9% and 72% of patients were current or former smokers, respectively). In addition, data on baseline ECOG Performance Status were reported for only 29% of the whole cohort (646/2,223).

**Table 1:** Design of the studies analysed and patient characteristics

Reference	Year	Study design	Patient characteristics						
			Total number	Age (years)		Gender, *n* (%)		Smoking (pack-years)	ECOG performance status, grade (%)
				Median	Range	Male	Female		
[18]	2015	Retrospective	62	57	23–83	44 (71)	18 (29)	ns	ns
[19]	2012	Retrospective	26	63	41–79	23 (89)	3 (11)	ns	ns
[20]	2017	Retrospective	122	61	ns	101 (83)	21 (17)	ns	ns
[21]	2014	Prospective	51	52	36–69	48 (94)	3 (6)	ns	ns
[22]	2013	Retrospective	70	52	37–86	66 (94)	4 (6)	ns	ns
[23]	2009	Retrospective	82	53.8	11–70	69 (84)	13 (16)	ns	ns
[24]	2012	Retrospective	88	59	26–83	74 (84)	14 (16)	ns	ns
[25]	2015	Retrospective	70	65	21–91	61 (87)	9 (13)	ns	ns
[26]	2016	Retrospective	78	62	24–79	63 (81)	15 (19)	ns	0/1 (98.8)
[27]	2014	Retrospective	108	67	43–85	93 (86)	15 (14)	ns	ns
[28]	2017	Retrospective	75	59	39–80	67 (89)	8 (11)	ns	ns
[29]	2014	Retrospective	214	58	ns	175 (82)	39 (18)	ns	ns
[30]	2017	Prospective	35	67.6	50–80	32 (91)	3 (9)	ns	0/1 (100)
[31]	2016	Retrospective	69	61	39–81	61 (88)	8 (12)	>10: 40 (60.9%)	ns
[32]	2015	Retrospective	72	60	39–75	61 (85)	11 (15)	>10: 41 (56.9%)	ns
[34]	2017	Retrospective	85	66	43–79	81 (95)	4 (5)	ns	ns
[35]	2011	Retrospective	47	55.1	15–86.1	39 (83)	8 (17)	ns	0/1 (95)
[36]	2016	Prospective	86	50	40–60	80 (93)	6 (7)	ns	ns
[37]	2013	Retrospective	81	65	34–81	74 (91)	7 (9)	ns	ns
[38]	2015	Retrospective	287	64	33–89	221 (77)	66 (23)	ns	0 (63)
[39]	2014	Retrospective	100	56	27–81	86 (86)	14 (14)	ns	ns
[40]	2015	Prospective	74	56	42–73	65 (88)	9 (12)	8.75 (median)	0/1 (100)
[42]	2016	Prospective	125	59	ns	93 (74)	32 (26)	ns	0/1 (100)
[43]	2014	Retrospective	40	48	21–69	30 (75)	10 (25)	ns	ns
[44]	2017	Retrospective	76	55	42–76	68 (89)	8 (11)	ns	ns

*ns* not stated

### Disease-related features

The most frequent primary tumour site was the oropharynx (1,150/2,223 patients, 51.7%), followed by the hypopharynx (377, 16.9%), larynx (345, 15.6%), nasopharynx (197, 8.9%), oral cavity (98, 4.4%), and others (56, 2.5%; Table 2). Information on HPV status was available for fewer than half of those with oropharyngeal tumour (508/1,150, 44.1%), and of these (as extrapolable from 7 of the 25 studies) 247 (48.6%) were p16/HPV-positive. Overall, in the majority of patients (1,709/1,799, 94.9%; no information available in four studies) the disease was in aggregated stage III/IV (Table 3).

**Table 2:** Disease features: tumour site

Reference	Number of patients	Oropharynx		Larynx	Hypopharynx	Oral cavity	Nasopharynx	Other
		Total	p16/HPV-positive					
[18]	62	14 (22%)	ns	10 (16%)	12 (20%)	3 (5%)	14 (22%)	9 (15%)
[19]	26	12 (46%)	ns	9 (35%)	2 (8%)	0	3 (11%)	0
[20]	122	122 (100%)	32 (26%)	0	0	0	0	0
[21]	51	20 (39%)	ns	0	21 (41%)	0	10 (20%)	0
[22]	70	70 (100%)	13 (19%)	0	0	0	0	0
[23]	82	13 (16%)	ns	0	6 (7%)	0	63 (77%)	0
[24]	88	58 (66%)	ns	15 (17%)	0	1 (1%)	7 (8%)	7 (8%)
[25]	70	25 (36%)	ns	0	36 (51%)	0	9 (13%)	0
[26]	78	47 (61%)	ns	3 (4%)	19 (24%)	5 (6%)	0	4 (5%)
[27]	108	28 (26%)	ns	29 (27%)	34 (31%)	17 (16%)	0	0
[28]	75	56 (75%)	ns	11 (15%)	5 (6%)	3 (4%)	0	0
[29]	214	135 (63%)	123 (57%)	40 (19%)	0	11 (5%)	0	28: (13%)
[30]	35	9 (26%)	ns	11 (31%)	12 (34%)	3 (9%)	0	0
[31]	69	41 (59%)	ns	20 (30%)	5 (7%)	3 (4%)	0	0
[32]	72	47 (66%)	ns	16 (22%)	6 (8%)	3 (4%)	0	0
[34]	85	0	ns	35 (41%)	50 (59%)	0	0	0
[35]	47	21 (45%)	ns	7 (15%)	4 (8%)	2 (4%)	13 (28%)	0
[36]	86	45 (52%)	ns	0	41 (48%)	0	0	0
[37]	81	0	ns	57 (70%)	24 (30%)	0	0	0
[38]	287	129 (45%)	ns	44 (15%)	55 (19%)	29 (10%)	30 (11%)	0
[39]	100	100 (100%)	14 (14%)	0	0	0	0	0
[40]	74	58 (78%)	25 (34%)	9 (12%)	7 (10%)	0	0	0
[42]	125	69 (56%)	37 (30%)	21 (17%)	11 (9%)	8 (6%)	8 (6%)	8 (6%)
[43]	40	0	0	0	0	0	40 (100%)	0
[44]	76	31 (41%)	3 (4%)	8 (10%)	27 (36%)	10 (13%)	0	0

*ns* not stated

**Table 3:** Disease features: stage

Reference	Tx	T1	T2	T3	T4	N0	N1	N2	N3	I	II	III	IV	III/IV
[18]	0	8	20	14	20	ns	ns	ns	ns	0	10 (16%)	18 (29%)	34 (55%)	52 (84%)
[19]	0	3	9	6	8	3	7	16	0	0	1 (3%)	7 (27%)	18 (70%)	25 (97%)
[20]	0	7	36	52	27	14	21	80	7	0	0	ns	ns	122 (100%)
[21]	ns	ns	ns	ns	ns	ns	ns	ns	ns	0	0	16 (31%)	35 (69%)	51 (100%)
[22]	0	0	0	20	50	ns	ns	ns	ns	0	8 (11%)	43 (52%)	19 (27%)	62 (89%)
[23]	0	22	25	17	18	10	22	43	7	4 (5%)	12 (15%)	30 (36%)	36 (44%)	66 (80%)
[24]	ns	ns	ns	ns	ns	ns	ns	ns	ns	3 (3%)	1 (1%)	15 (16%)	70 (80%)	85 (96%)
[25]	0	0	34	16	20	29	10	18	13	ns	ns	ns	ns	ns
[26]	ns	ns	ns	ns	ns	ns	ns	ns	ns	ns	ns	23 (30%)	55 (70%)	78 (100%)
[27]	0	26	37	13	32	51	14	40	3	18 (17%)	20 (18%)	19 (18%)	51 (47%)	70 (65%)
[28]	0	6	31	27	11	0	11	59	5	0	0	10 (13%)	65 (87%)	75 (100%)
[29]	ns	ns	ns	ns	ns	ns	ns	ns	ns	ns	ns	ns	ns	ns
[30]	0	0	13	16	6	13	4	16	2	0	4 (11%)	10 (29%)	21 (60%)	31 (89%)
[31]	0	4	28	27	10	16	10	39	4	0	6 (9%)	18 (26%)	45 (65%)	63 (91%)
[32]	0	6	25	31	10	9	11	47	5	0	0	18 (25%)	54 (75%)	72 (100%)
[34]	0	0	19	49	17	26	15	44	0	0	0	33 (39%)	52 (61%)	85 (100%)
[35]	ns	ns	ns	ns	ns	ns	ns	ns	ns	0	2 (4%)	11 (23%)	34 (73%)	45 (96%)
[36]	ns	ns	ns	ns	ns	ns	ns	ns	ns	0	0	4 (5%)	82 (95%)	86 (100%)
[37]	0	2	11	43	25	28	15	38	3	0	0	32 (39%)	49 (61%)	81 (100%)
[38]	0	32	92	78	85	30	32	190	35	0	0	54 (19%)	233 (81%)	287 (100%)
[39]	0	14	39	23	24	4	12	80	4	ns	ns	ns	ns	ns
[40]	0	0	29	26	19	0	0	ns	6	0	0	ns	ns	74 (100%)
[42]	7	ns	ns	ns	ns	0	0	119	6	0	0	ns	ns	125 (100%)
[43]	0	14	ns	ns	ns	0	19	ns	ns	7	ns	ns	ns	ns
[44]	0	2	10	27	37	12	7	54	3	1 (1%)	1 (1%)	11 (15%)	63 (83%)	74 (98%)

*ns* not stated

### Treatment-related features

Most patients (1,467/1,544, 95%) were treated with IMRT, while 77 (5%) received 3D-conformal radiotherapy (3DCRT). No information on the radiotherapy technique used was available in seven studies (Table 4). The most adopted radiotherapy regimen consisted of conventional fractionation of 1.8 or 2 Gy per fraction for a total dose of 66–72 Gy in the majority of cases (22/23 papers; no available data in two studies). Concurrent chemoradiotherapy was the most frequent treatment schedule in our analysis, being used in 1,562/2,009 patients (77.7%; no available information in only one study). Standard three-weekly 100 mg/m^2^ cisplatin was the chosen regimen in almost half of the included studies (11/24). Finally, a very small group of patients received induction chemotherapy before radiotherapy (181/2,223, 8.1%) in seven studies. On the basis that these studies were not excluded by our entry search criteria, they were retained in the analysis.

**Table 4:** Treatment-related features

Reference	Radiotherapy					Induction chemotherapy			Concurrent chemotherapy		
	Number of patients		Regimen^a^	Dose (Gy)		Number of patients	Type of chemotherapy	No. of cycles (median)	Number of patients	Type of chemotherapy	No. of cycles (median)
	3DCRT	IMRT		Total (median)	Per fraction						
[18]	32	30	Conventional	70	2	20	Docetaxel/cisplatin	ns	35	Cisplatin 40 mg/m^2^ every 7 days	6
[19]	0	26	Conventional	70	1.8	0	Not administered	0	26	Cetuximab	ns
[20]	0	122	Conventional	70	2	0	Not administered	0	122	Cisplatin 100 mg/m^2^ every 21 days	ns
[21]	0	51	Conventional	70	ns	0	Not administered	0	51	Cisplatin 100 mg/m^2^ every 21 days	ns
[22]	ns	ns	Conventional	72	2	0	Not administered	0	44	Cisplatin 100 mg/m^2^ every 21 days	ns
[23]	ns	ns	Conventional	72	2	0	Not administered	0	68	Cisplatin 100 mg/m^2^ every 21 days	ns
[24]	0	88	Conventional	70	2	0	Not administered	0	74	Cisplatin + 5-fluorouracil	ns
[25]	0	70	Conventional	66	1.8	0	Not administered	0	70	Cisplatin 100 mg/m^2^ every 21 days	ns
[26]	ns	ns	Conventional	70	2	0	Not administered	0	70	Other	ns
[27]	0	108	Conventional	70	2	48	Docetaxel/cisplatin	2	44	Cisplatin + 5-fluorouracil	6
[28]	0	75	Conventional	70	2	0	TPF	1	75	Cisplatin 100 mg/m^2^ every 21 days	ns
[29]	ns	ns	ns	ns	ns	0	Not administered	ns	ns	ns	ns
[30]	40	0	Conventional	70	2	0	Not administered	0	35	Cisplatin 30 mg/m^2^ every 7 days	ns
[31]	0	69	Conventional	70	2	15	ns	ns	46	Cisplatin 40 mg/m^2^ every 7 days	ns
[32]	0	72	Conventional	70	2	15	ns	ns	40	Cisplatin 30 mg/m^2^ every 7 days	6
[34]	ns	ns	Conventional	66	2	0	Not administered	0	85	Other	6
[35]	5	42	Conventional	66	2	0	Not administered	0	47	Cisplatin + 5-fluorouracil	ns
[36]	0	86	Conventional	72	2	0	Not administered	0	86	Other	ns
[37]	ns	ns	Conventional	70	2	0	Not administered	0	47	Cisplatin + 5-fluorouracil	ns
[38]	0	287	Conventional	66	2	26	ns	ns	86	Other	ns
[39]	0	100	Accelerated	69.96	2.12	0	Not administered	0	100	Cisplatin 100 mg/m^2^ every 21 days	ns
[40]	ns	ns	ns	ns	ns	0	Not administered	0	74	Cisplatin 100 mg/m^2^ every 21 days	ns
[42]	0	125	Conventional	70	2	0	Not administered	0	125	Cisplatin 100 mg/m^2^ every 21 days	ns
[43]	0	40	Conventional	70.2	1.8	40	Docetaxel/cisplatin	3	36	Cisplatin 100 mg/m^2^ every 21 days	ns
[44]	0	76	Conventional	72	2	0	Not administered	0	76	Cisplatin 100 mg/m^2^ every 21 days	ns

*ns* not stated, *3DCRT* 3-D conformal radiotherapy, *IMRT* intensity-modulated radiotherapy, *TPF* docetaxel, cisplatin, 5-fluorouracil

^a^Conventional: conventional once-daily fractionation. Accelerated: accelerated fractionation

### Prognostic impact of FDG PET: descriptive analysis

The timing of FDG PET was different among the studies included in the analysis (Table 5). A single baseline assessment time-point was present in almost half of the studies (12/25) while a combination of pretreatment, interim (during treatment) and posttreatment scans was described in four (pretreatment plus interim), seven (pretreatment plus posttreatment) and two (pretreatment plus interim plus posttreatment) studies. Among those studies providing data on more than a single time-point, a time-weighted analysis exploring changes over time (“delta”) of specific metabolic semiquantitative or qualitative features was additionally reported in seven. As a single variable, MTV and SUV_max_ were the main metabolic parameters addressed in nine and seven studies, respectively. A qualitative analysis was used in three studies [29, 31, 42]. Zschaeck et al. [44] determined SUV_mean_ in irradiated normal mucosa tissue to explore the impact of off-target hypermetabolism and its change over time. Only a limited number of alternative prognostic biomarkers (comparators) were reported in parallel with the metabolic evaluation (nine studies). The median overall follow-up time for all studies was 23.6 months (range 15–55.8 months). In terms of threshold or cut-off values to discriminate worse from better outcomes, a large variability was observed for each intervention. Finally, a large heterogeneity characterized the prognostic information which could be extracted from each paper.

**Table 5:** Prognostic impact of FDG PET: descriptive analysis

Reference	Timing of FDG PET	Main metabolic parameter	Significant threshold/cut-off value	Prognostic comparator	Median follow-up (months)	Primary outcome measure	Main message
[18]	Pretreatment	MTV	14 ml	ns	18	LRC, PFS	3-year LRFS and DFS lower in patients with MTV ≥14 ml
[19]	Pretreatment Interim Posttreatment Analysis of change over time	SUV_max_	ns	ns	29.2	PFS, DSS	Metabolic response on posttreatment PET correlated with 2-year PFS and DSS
[20]	Pretreatment	MTV	ns	T stage, N stage, HPV status	30.5	LRC, OS	PET-based nomogram: MTV as a continuous variable correlated with 2-year OS
[21]	Pretreatment Interim Analysis of change over time	SUV_max_	ns	ns	23	DFS, OS	SUV_max_ reduction ratio <0.64 associated with inferior 2-year OS and DFS
[22]	Pretreatment	TLG	121.9 g	Uniformity (texture), HPV status	27	PFS, DSS, OS	TLG >121.9 g and uniformity ≤0.138 associated with inferior PFS, DSS and OS
[23]	Pretreatment	MTV	40 ml	ns	ns	DFS	Worse short-term outcome and shorter DFS in patients with MTV >40 ml
[24]	Pretreatment	SUV_mean_	ns	ns	15	DFS	SUV_mean_ >7 (median of cohort) correlated with inferior 2-year DFS
[25]	Pretreatment Posttreatment	SUV_max_	5	Haemoglobin level	38.4	LRC, OS	Posttreatment SUV_max_ <5 and pretreatment haemoglobin >12 g/dl correlated with superior LRC and OS
[26]	Pretreatment Posttreatment	SUV_max_	4.4	ns	52.7	PFS, OS	Posttreatment SUV_max_ <4.4 correlated with superior 3-year PFS and OS
[27]	Pretreatment	MTV, uptake pattern	20 ml	ns	36.4	DFS, DSS	MTV >20 ml and qualitative uptake pattern (ring-shape) correlated with inferior DFS and DSS
[28]	Pretreatment Interim Analysis of change over time	TLG, MTV	2.95 g/ml	ns	28	LRC, DFS, OS	Index node SUV_mean_ on interim PET <2.95 g/ml and TLG, MTV reduction >50% on interim PET correlated with superior LRC, DFS and OS
[29]	Pretreatment Posttreatment Analysis of change over time	Hopkins five-point scale	ns	HPV status	27	PFS, OS	Hopkins five-point qualitative response interpretation and HPV status able to discriminate PFS and OS
[30]	Pretreatment Posttreatment Analysis of change over time	SUV_max_	ns	ns	ns	LRC	SUV_max_ reduction ratio <1.04 associated with inferior LRC
[31]	Pretreatment Interim Posttreatment Analysis of change over time	Visual grading	ns	ns	28	LRC, DFS, OS	Visual grading response interpretation able to discriminate 2-year LRC, DFS and OS
[32]	Pretreatment, Interim	TLG	9.4 g	ns	25	LRC, DFS, OS	TLG on interim PET <9.4 g correlated with superior 2-year LRC, DFS and OS
[34]	Pretreatment	MTV	28.7 ml	T stage	ns	LRC, OS	MTV >28.7 ml correlated with inferior 3-year LRC and OS
[35]	Pretreatment Posttreatment	MTV	ns	ns	34	DFS, OS	Increase in MTV2.0 (volume above SUV threshold of 2) of 21 ml associated with inferior DFS and OS
[36]	Pretreatment	SUV_max_	19.4	*k*_ep_-tumour, *v*_e_-node (MR parameters)	28	PFS, OS	*k*_ep_-tumour,* v*_e_-node, SUV_max_ independently able to discriminate 3-year PFS and OS
[37]	Pretreatment	MTV	18 ml	Primary tumour site	40.4	LRC, OS	MTV >18 ml correlated with inferior 3-year LRC and OS
[38]	Pretreatment	SUV_max_	13	Gross tumour volume, cisplatin delivery, smoke	32	DFS, OS	SUV_max_ <13 (median of cohort) correlated with superior DFS and OS
[39]	Pretreatment	MTV	9.7 ml	ns	55.8	LRC, PFS, OS	MTV <9.7 ml (median of cohort) correlated with superior 5-year LRC, PFS and OS
[40]	Pretreatment Posttreatment	MTV	ns	ns	50	LRC, PFS	Pretreatment MTV above the median correlated with inferior LRC and PFS
[42]	Pretreatment Interim Analysis of change over time	Hopkins five-point scale	ns	HPV status	20.4	PFS, OS	Hopkins five-point qualitative response interpretation and HPV status able to discriminate PFS and OS
[43]	Pretreatment	MTV	ns	ns	32.5	OS	MTV3.0 (volume above a SUV threshold of 3) >23.01 ml associated with inferior OS
[44]	Pretreatment Interim	SUV_mean_ (in MST)	ns	ns	29.3	LRC, OS	SUV_mean_ of MST on interim PET >2.3 g/ml (median of cohort) correlated with superior LRC and OS

*ns* not stated, *MST* mucosa and submucosa soft tissues, k_*ep*_ efflux rate constant of primary tumour on dynamic contrast-enhanced MR imaging, v_*e*_ relative volume of extracellular extravascular space of largest metastatic lymph node on diffusion-weighted MR imaging, *LRC* locoregional control, *PFS* progression-free survival, *DSS* disease-specific survival, *OS* overall survival, *DFS* disease-free survival

### Prognostic impact of pretreatment FDG PET: meta-analysis

**Fig. 2:**
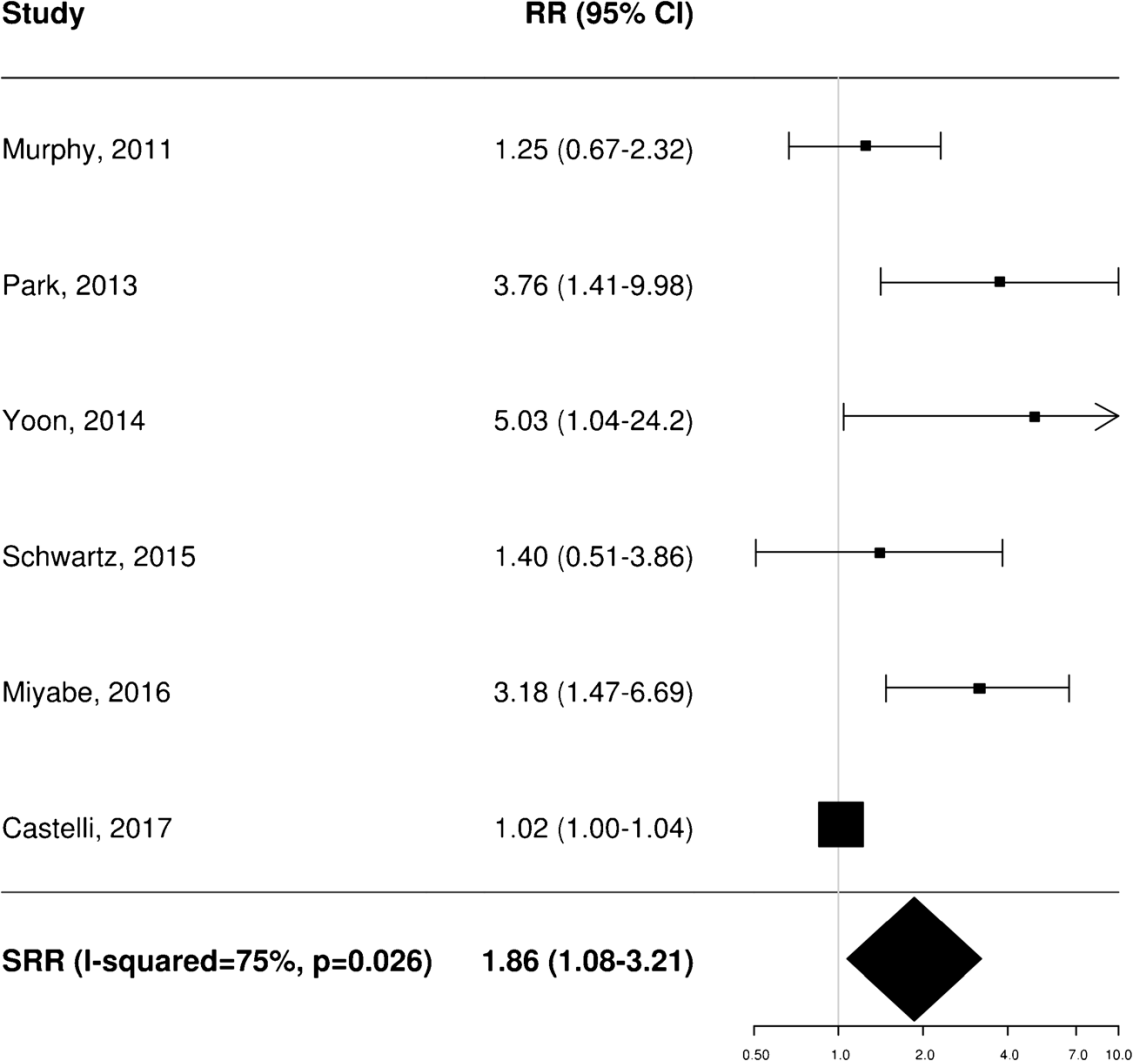
Impact of pretreatment MTV on overall survival

**Fig. 3:**
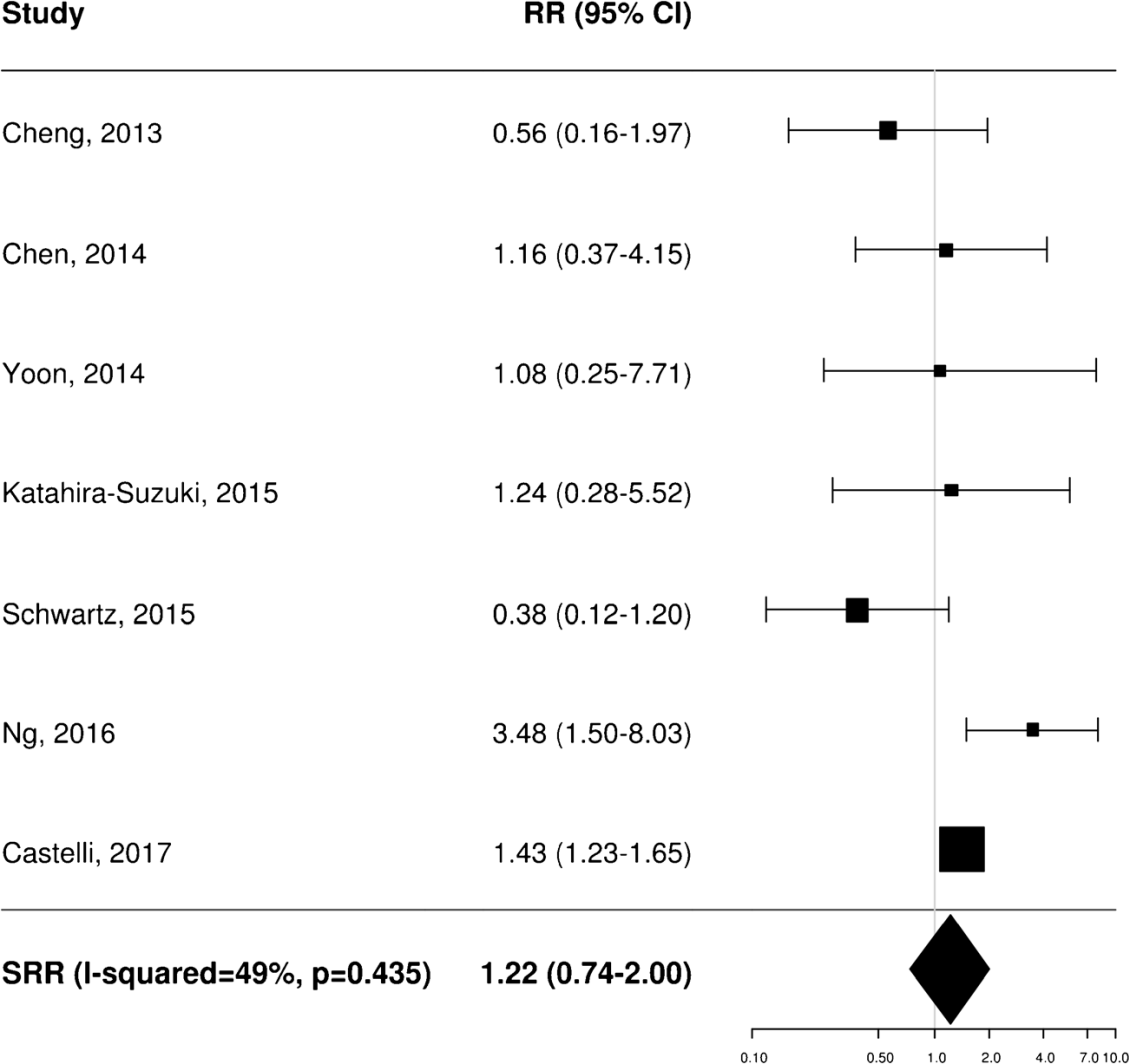
Impact of pretreatment SUV_max_ on overall survival

**Fig. 4:**
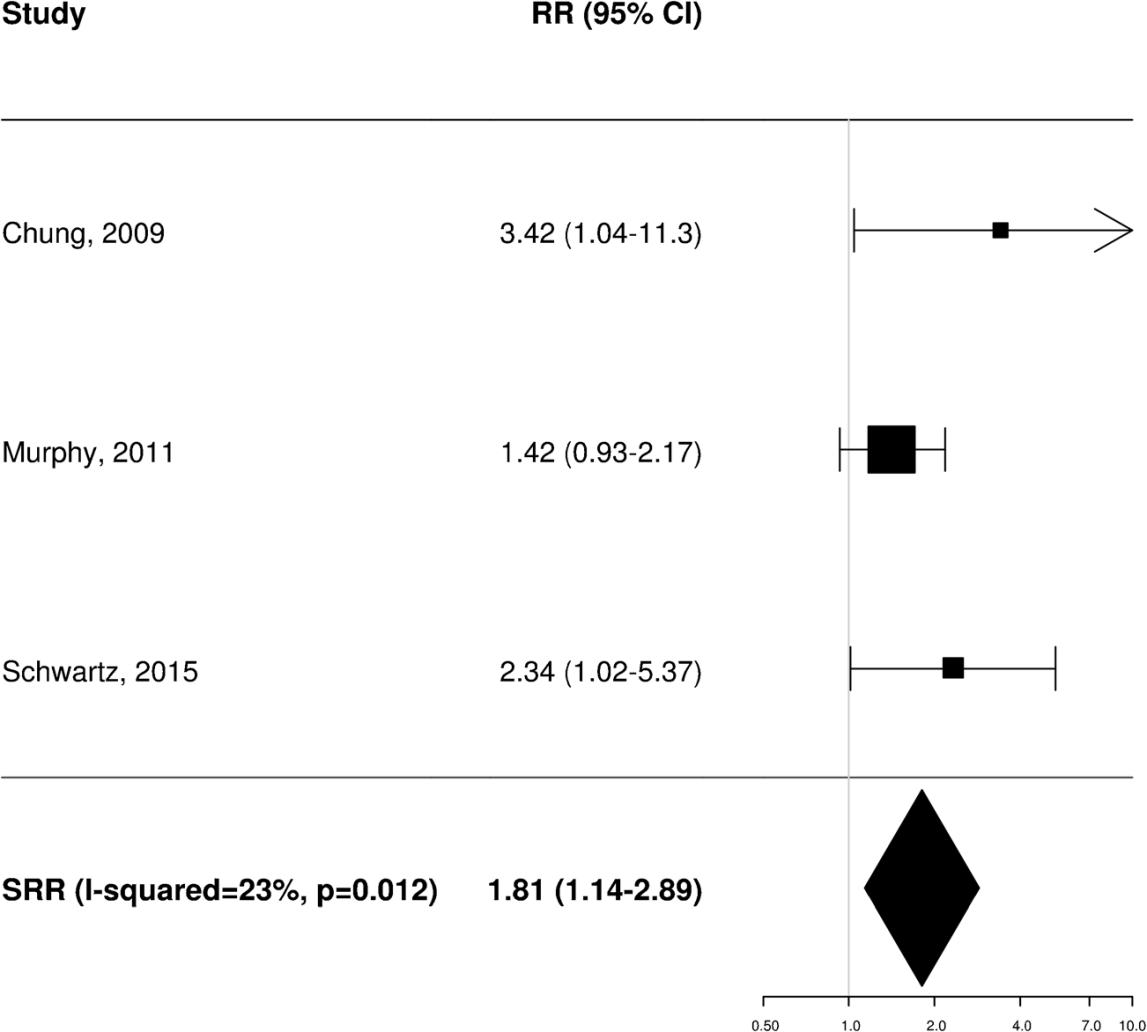
Impact of pretreatment MTV on progression-free survival

**Fig. 5:**
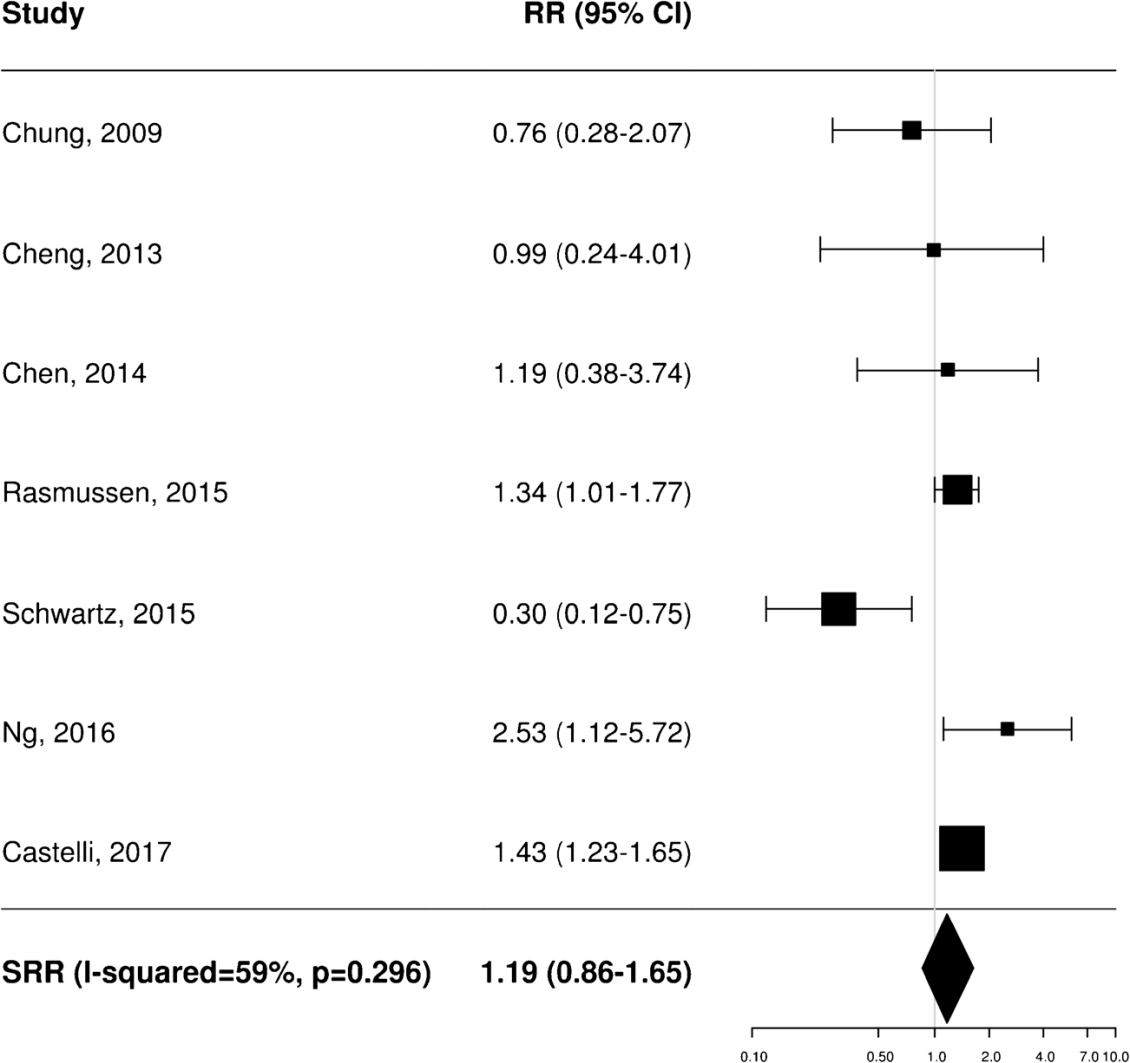
Impact of pretreatment SUV_max_ on progression-free survival

**Fig. 6:**
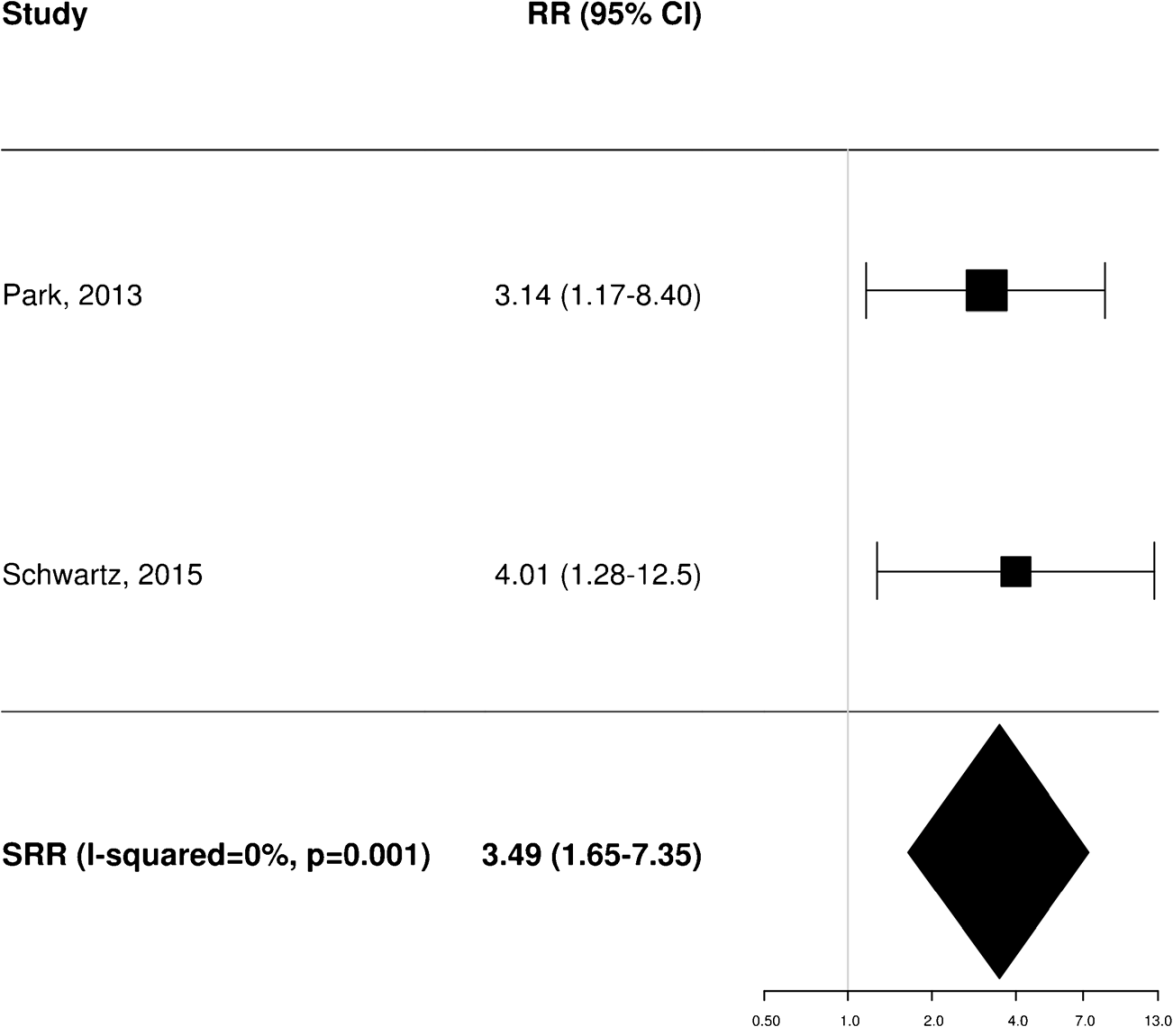
Impact of pretreatment MTV on locoregional control

**Fig. 7:**
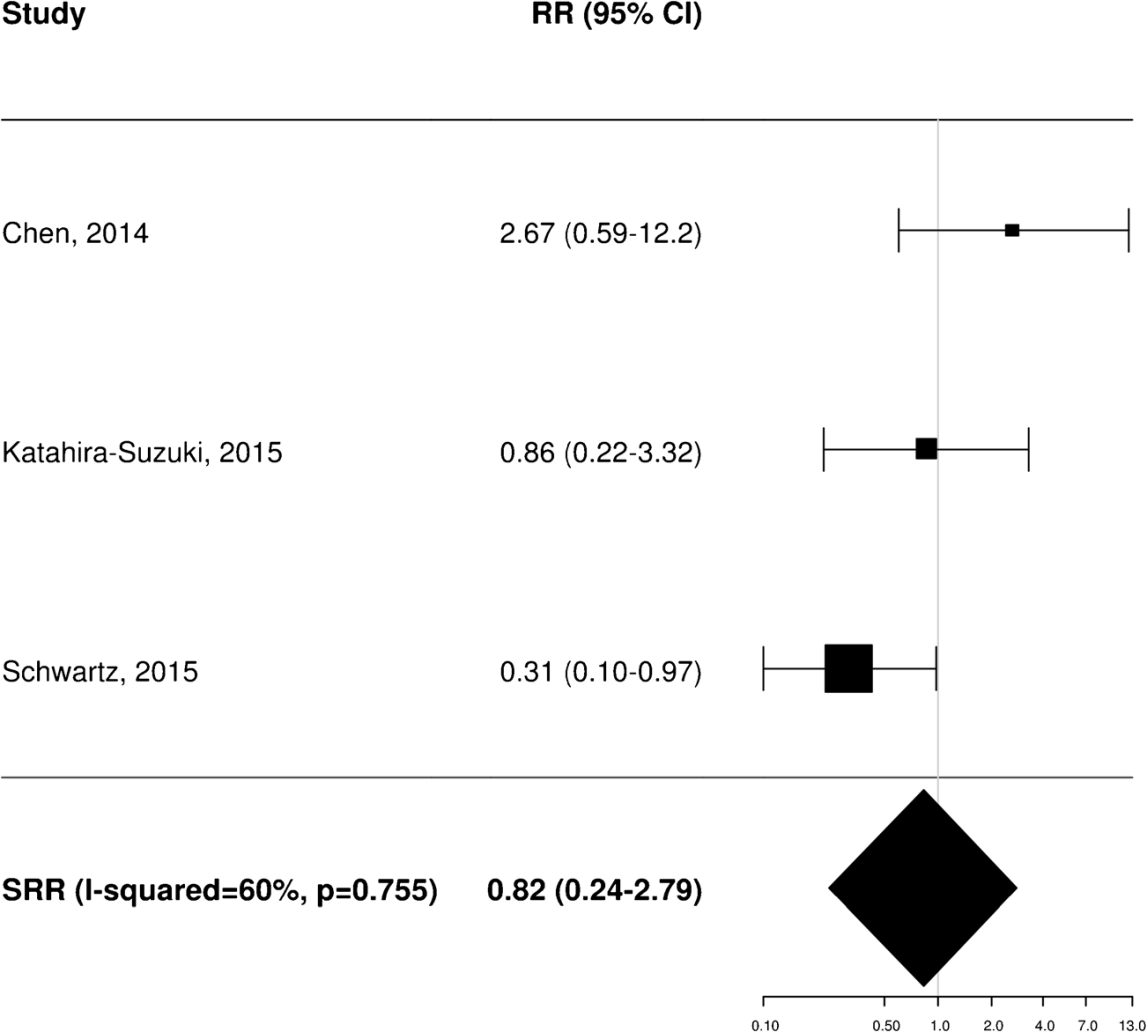
Impact of pretreatment SUV_max_ on locoregional control

A total of 11 [20–22, 25, 34–37, 40, 43, 45], 8 [20–23, 35, 36, 38, 40] and 4 [21, 25, 37, 40] independent studies provided RR estimates for the association between baseline FDG PET and OS, PFS and LRC, respectively. These studies were thus included in the meta-analysis aiming to assess the potential prognostic impact of pretreatment metabolic features on patient outcome. Of note, Castelli et al. [45] performed a secondary analysis in the same patient population analysed in a previous work [20] providing additional data with distinct RR estimates that were therefore worthy of inclusion. Instead, the results reported by Zschaeck et al. [44] were not considered, since the prognostic value of uptake in mucosa soft tissue was not investigated in any of the other included studies. In terms of baseline FDG PET parameters, the analysis was limited to MTV and SUV_max_, for which there were six and seven RR estimates for OS (Figs. 2 and 3), three and seven for PFS (Figs. 4 and 5), and two and three for LRC (Figs. 6 and 7), respectively. Higher MTV values for the primary or primary and nodal disease combined were significantly associated with a worse OS (SRR 1.86, 95% CI 1.08–3.21), PFS (SRR 1.81, 95% CI 1.14–2.89) and LRC (SRR 3.49, 95% CI 1.65–7.35), Instead, we found no statistically significant association between SUV_max_ and any of OS, PFS and LRC. Given the large between-study heterogeneity (*I*^2^ > 50%) that affected the summary measures, no effort was made to define a cumulative threshold value for an unfavourable prognosis. Finally, apart from an unclear or high risk of bias in terms of patient selection (21/25 studies, 84%) because of the predominantly retrospective nature of the included studies, according to the QUADAS-2 tool [46] the overall quality was good with low risks of bias and concerns regarding applicability in the remaining domains (Supplementary Table 6).

## Discussion

In the era of precision oncology, the lack of prognostic biomarkers has hindered the evolution of standard-of-care management in HNSCC. Apart from HPV status, no molecular stratification is currently available for use in daily practice. In the last two decades, steady technological progress has highlighted the potential of imaging as a comprehensive tumour biomarker [47]. In the field of functional imaging, FDG PET is the most widespread, easily accessible modality that is able to provide surrogate metabolic information on tumour burden. The aim of our work was to define whether distinct FDG PET features can be intrinsically associated with prognostic relevance in the context of nonmetastatic HNSCC. We acknowledge several limitations which have to be taken into account when interpreting the data presented. First, most studies included in our systematic review were retrospective. Although a strict search methodology was followed, their potential heterogeneity in terms of patient selection, treatment administration and outcome measures may have affected the consistency of our analysis. Second, the technical variability in the performance of FDG PET scans is also a factor that cannot be ignored with a retrospective study design; only a prospective design can ensure that consensus acquisition recommendations [14] are rigorously adopted. Third, among the included studies the methods used to calculate the FDG PET metrics were not consistent. Heterogeneity in their definition has to be taken into account particularly for SUV_max_ and MTV, for which several threshold values were shown to be significant in discriminating patients with different outcomes.

Renewed interest in the role of FDG PET in the management of HNSCC was recently prompted by the publication of the PET-NECK trial [11]. The findings of this large prospective, multicentre phase 3 trial are practice-changing, since the study provided definitive evidence in favour of a response evaluation centred on the high negative predictive value (NPV) of a 12-week posttreatment FDG PET scan. However, the study had two main limitations that prevented the clarification of other relevant issues on the role of FDG PET in the management of HNSCC. First, none of the 564 patients enrolled in the trial underwent a baseline FDG PET scan; a qualitative comparison between pretreatment and posttreatment scans was therefore not performed. Second, FDG PET semiquantitative metrics could not be evaluated due to nonuniform calibration among the different scanners. From this perspective, the PET-NECK trial did not add any new data to the available low-level body of evidence on the prognostic role of specific FDG PET semiquantitative and qualitative features in HNSCC. Although many investigators have focused on this topic in the last 15 years [48], the literature is characterized by inconclusive and heterogeneous findings [49].

A crucial aspect that needs again to be underlined is the strict dependence of FDG PET information on the image acquisition modality, which in turn may be influenced by a series of factors, ranging from the technical parameters of the scanner to the timing of the scan with respect to treatment. As also demonstrated in our descriptive analysis (Table 5), there is significant variability in the correlation between semiquantitative metrics and outcome measures in HNSCC. We have already pointed out that in the posttreatment scenario a negative PET scan at 12 weeks after chemoradiation is a prognostic biomarker of long-term complete remission based on level 1 evidence. However, standardized interpretation of response to treatment is lacking. In this context, the Hopkins criteria are the only proposed scoring system for qualitative interpretation of FDG PET in HNSCC. Marcus et al. [29] showed that a five-point scale based on prespecified qualitative descriptors is accurate in discriminating complete from incomplete responses. The application of the Hopkins criteria resulted in a high NPV of 91.1% with an overall diagnostic accuracy of 86.9%. Notably, the results of the ECLYPS study [42] prospectively confirmed the reliability of the Hopkins criteria applied 12 weeks after the end of treatment, with an overall NPV of 92.1% and a very low number of equivocal reports. As accurately described by Garibaldi et al. [50] in a recent systematic review, the potential prognostic and predictive relevance of an interim FDG PET scan (scan acquired during treatment) is a controversial matter. At present, no firm conclusions can be drawn as to the ideal metabolic parameter to analyse early in treatment, the most informative threshold value, or the best time to re-scan the patient.

Taking all together, the use of FDG PET in patients with HNSCC provides prognostic information through standardized qualitative assessment at a minimum of 12 weeks after chemoradiation, but no added value during its delivery. It is therefore a rational approach to investigate before treatment whether baseline semiquantitative metrics are intrinsically able to characterize the outcome in patients with locally advanced disease. Conflicting evidence is available from the literature. Pak et al. [51] performed a systematic review and meta-analysis of 13 studies (1,180 patients) to assess the prognostic role of MTV and TLG before treatment. The authors found that high values of both volumetric parameters correlated significantly with a worse outcome. The pooled HRs for OS were 3.51 (95% CI 2.62–4.72, *p* < 0.00001) and 3.14 (95% CI 2.24 – 4.40, *p* < 0.00001) for MTV and TLG, respectively. However, the generalizability of these results is open to question. First, loose criteria were followed in the literature search strategy and inclusion of articles. Second, for both parameters no threshold values portending a worse outcome were defined, thus preventing further analysis of the data.

In a prospective study in 77 patients affected by stage II–IV HNSCC, Schinagl et al. [52] consistently applied five different segmentation methods for coregistered CT and FDG PET scans at baseline. Among the different metrics obtained, only the gross tumour volume (GTV) visually delineated on FDG PET images was significantly correlated with outcome in oral cavity and oropharyngeal tumours, while all isocontour-based volumes, SUV_mean_ and SUV_max_, were not. A large single-centre [38] retrospective study on 287 patients receiving IMRT-based treatment showed different results. In a univariate analysis, increasing values of SUV_max_ (as a logarithmic variable) yielded a HR of 1.72 (95% CI 1.34–2.19) for a worse disease-free survival (DFS) and OS. Multivariate analysis showed an additive effect of increasing GTV (HR 1.74, 95% CI 1.33–2.27; increase in interquartile range from 25% to 75% corresponding to an increase in GTV from 27.4 cm^3^ to 95.8 cm^3^) and increasing SUV_max_ (HR 1.34, 95% CI 1.01–1.77; increase in interquartile range from 25% to 75% corresponding to an increase in SUV_max_ from 9.6 to 16.8) for a worse prognosis.

The link between FDG avidity and tumour volume has been further explored by different groups focusing on MTV. In this regard, the correlative, prospective imaging study of the randomized phase 3 RTOG 0522 trial [40] is noteworthy. Of the whole sample of 940 patients enrolled, 74 from 19 different centres provided both pretreatment and posttreatment FDG PET scans, as mandated upon inclusion. A prespecified acquisition imaging protocol was followed in all patients. Excellent centralized interobserver agreement (intraclass correlation coefficient ≥0.80) on semiquantitative metrics was reported. Based on voxels with a minimum of 40% SUV_max_, baseline primary MTV above the median was the strongest prognosticator of worse LRC (HR 4.01, 95% CI 1.28–12.52, *p* = 0.2). Other retrospective studies [23, 27, 37] have underlined the prognostic value of baseline MTV, reporting different cut-off values as most significant for a worse outcome (combined primary and nodal MTV >40 ml, >20 ml and >18 ml correlating with worse DFS [23], LRC and OS [27], and disease-specific survival [37], respectively). The prognostic value of MTV analysed as a continuous variable has also been reported.

In a single-centre retrospective analysis in 83 patients, Tang et al. [53] found that an increase in primary baseline MTV of 17 ml (from the 25th to the 75th percentile) was associated with a doubling of the risk of disease progression (*p* = 0.0002) and of death (*p* = 0.0048). Of note, combined primary and nodal MTV (as a continuous variable) was also associated with a shorter PFS (HR 4.23, *p* < 0.0001; CI not reported) and OS (HR 3.21, *p* < 0.0029; CI not reported) in the subgroup of 64 patients with p16-positive oropharyngeal cancer. In a larger cohort of 122 patients with oropharyngeal cancer, Castelli et al. [45] assessed whether the use of different absolute and relative thresholds of SUV_max_ result in different discriminatory power of MTV. Using a 51% relative SUV_max_ threshold, combined primary and nodal MTV was the only significant factor in a multivariate analysis predicting OS (HR 1.43 per 10 ml, CI 1.23–1.65, *p* < 0.001) and DFS (HR 1.43 per 10 ml;,CI 1.23–1.65, *p* = 0.03). The optimal cut-off value for MTV 51% was 22.7 ml, which was able to discriminate 2-year DFS with rates of 63.3% versus 32.9% and LRC with rates of 68% versus 35.3%.

The absence of a consensus methodology on VOI delineation is clearly a limitation when comparing different datasets on the prognostic relevance of MTV, since no single optimal cut-off value is recognized. In line with previous experience, our data reinforce the prognostic role of pretreatment MTV as the most informative semiquantitative metabolic feature. In line with our search inclusion criteria, the patient population analysed was extremely homogeneous (about 95% of the whole sample size) in terms of disease stage, radiotherapy technique used and schedule of concomitant chemoradiotherapy. With all due limitations, our analysis provides further evidence on the predominant impact of pretreatment MTV on HNSCC outcome compared with all other available FDG PET metrics. Further consideration of its role also as a predictive biomarker may be generated by pattern-of-failure data correlating baseline FDG PET and radiation dose distribution in HNSCC. Due et al. [54] performed a retrospective analysis in 304 HNSCC patients with the aim of correlating the pattern of disease failure with FDG uptake on pretreatment PET scans. By performing a deformable registration of CT scans acquired at the time of recurrence with the planning PET/CT scan, the authors showed that 96% of relapses (95% CI 86–99%) occurred in the high-dose region. In addition, they found that recurrence density was higher in the central part of the target volume (*p* < 0.0001), with a significant correlation with increasing FDG avidity (*p* = 0.036). In a smaller cohort of 44 patients enrolled in a prospective phase 2 trial, Leclerc et al. [55] showed that all ten recurrences arose in areas receiving >95% of the dose determined on PET-based plans. A similar finding was reported by Mohamed et al. [56], who hypothesized that a 1-cm margin in addition to the 50% SUV_max_ isocontour on pretreatment FDG PET scans would cover the majority of type A recurrences (according to the authors’ definition, those that arise in the central high-dose area).

Once again, it has to be underlined that, among others, the main limitations of FDG in HNSCC are its suboptimal specificity and the large variability in segmentation methods. Potentially, it could be hypothesized that hypoxia PET [57] and diffusion-weighted magnetic resonance imaging [58] may be more refined imaging biomarkers in the field of HNSCC. However, conclusive results on their prognostic impact have long been awaited, mainly due to the lack of reproducibility and cost issues preventing their adoption on a large scale. In our opinion FDG PET will remain the most widespread functional imaging modality used in clinical practice for many years to come.

### Conclusion

The absence of prognostic biomarkers is a critical limitation in the management of locally advanced HNSCC. With all due limitations, our analysis showed that MTV defined from pretreatment FDG PET scans has the strongest impact on patient outcome after standard concurrent chemoradiotherapy. Prospective studies to corroborate this finding through standardized FDG PET acquisition and segmentation methods are warranted.

## Electronic supplementary material

ESM 1 (DOCX 68 kb)

ESM 2 (DOCX 174 kb)

ESM 3 (DOCX 21 kb)
