# Supplementary material for: What is the prognostic impact of FDG PET in locally advanced head and neck squamous cell carcinoma treated with concomitant chemo-radiotherapy? A systematic review and meta-analysis
Source: Eur J Nucl Med Mol Imaging. 2018 Jun 9;45(12):2122–38. doi: 10.1007/s00259-018-4065-5 (PMC6182396; doi:10.1007/s00259-018-4065-5)
Supplement: Supplementary file 1 — (DOCX 68 kb) [file 259_2018_4065_MOESM1_ESM.docx]

**1. MEDLINE search strategy**

("head and neck neoplasms"[MeSH Terms] OR "head and neck neoplasms"[All Fields]) AND ("chemoradiotherapy"[MeSH Terms] OR "chemoradiotherapy"[All Fields]) AND ("positron emission tomography computed tomography"[MeSH Terms] OR "positron emission tomography computed tomography"[All Fields]) AND (("2007/01/01"[PDAT] : "2017/02/28"[PDAT]) AND English[lang])

**2. Embase search strategy**

'head and neck cancer'/exp OR 'ent cancer' OR 'orl cancer' OR 'cancer, head and neck' OR 'cervicofacial cancer' OR 'ear nose throat cancer' OR 'head and neck cancer' OR 'head neck cancer' OR 'otorhinolaryngeal cancer' OR 'otorhinolaryngologic cancer' OR 'otorhinolaryngological cancer' AND ('chemoradiotherapy'/exp OR 'chemoradiation' OR 'chemoradiotherapy' OR 'radiochemotherapy') AND ('computer assisted emission tomography'/exp OR 'computer assisted emission tomography' OR 'positron-emission tomography and computed tomography' OR 'positron-emission tomography/computed tomography')
