# Supplementary material for: What is the prognostic impact of FDG PET in locally advanced head and neck squamous cell carcinoma treated with concomitant chemo-radiotherapy? A systematic review and meta-analysis
Source: Eur J Nucl Med Mol Imaging. 2018 Jun 9;45(12):2122–38. doi: 10.1007/s00259-018-4065-5 (PMC6182396; doi:10.1007/s00259-018-4065-5)
Supplement: Supplementary file 2 — (DOCX 174 kb) [file 259_2018_4065_MOESM2_ESM.docx]

**Reference list of eligible papers and reason for exclusion from systematic review**

1. Abgral, Ronan, Pierre-Yves Le Roux, Nathalie Keromnes, Jean Rousset, Gérald Valette, Dominique Gouders, Cyril Leleu, et al. “Early Prediction of Survival Following Induction Chemotherapy with DCF (Docetaxel, Cisplatin, 5-Fluorouracil) Using FDG PET/CT Imaging in Patients with Locally Advanced Head and Neck Squamous Cell Carcinoma.” European Journal of Nuclear Medicine and Molecular Imaging 39, no. 12 (December 2012): 1839–47. doi:10.1007/s00259-012-2213-x.– limited patients’ population
2. Castelli J, Depeursinge A, De Bari B, Devillers A, De Crevoisier R, Bourhis J, et al. Metabolic tumor volume and total lesion glycolysis in oropharyngeal cancer treated with definitive radiotherapy which threshold is the best predictor of local control? Clin Nucl Med 2017;42:e281–5. doi:10.1097/RLU.0000000000001614 – duplicate of paper already included
3. Chaput A. “EANM’16. High FDG Uptake on Pre-Radiotherapy PET/CT and Preferential Sites of Local Relapse after Chemoradiotherapy for Locally Advanced Head and Neck Cancer.” European Journal of Nuclear Medicine and Molecular Imaging 43, no. S1 (October 2016): 1–734. doi:10.1007/s00259-016-3484-4 – RT planning study
4. Due, Anne K., Ivan R. Vogelius, Marianne C. Aznar, Søren M. Bentzen, Anne K. Berthelsen, Stine S. Korreman, Annika Loft, Claus A. Kristensen, and Lena Specht. “Recurrences after Intensity Modulated Radiotherapy for Head and Neck Squamous Cell Carcinoma More Likely to Originate from Regions with High Baseline [18F]-FDG Uptake.” Radiotherapy and Oncology 111, no. 3 (June 2014): 360–65. doi:10.1016/j.radonc.2014.06.001. – RT planning study
5. Hoshikawa, Hiroshi, Terushige Mori, Yuka Yamamoto, Takehito Kishino, Takashi Fukumura, Yasushi Samukawa, Nozomu Mori, and Yoshihiro Nishiyama. “Prognostic Value Comparison Between 18F-FLT PET/CT and 18F-FDG PET/CT Volume-Based Metabolic Parameters in Patients with Head and Neck Cancer:” Clinical Nuclear Medicine 40, no. 6 (June 2015): 464–68. doi:10.1097/RLU.0000000000000652.– main investigation on tracer different than FDG (FLT PET)
6. Jeong, Y., S. Baek, J.W. Park, J.H. Joo, S.J. Kim, and S.-W. Lee. “Lymph Node Standardized Uptake Values at Pre-Treatment 18F-Fluorodeoxyglucose Positron Emission Tomography as a Valuable Prognostic Factor for Distant Metastasis in Nasopharyngeal Carcinoma.” British Journal of Radiology 90, no. 1071 (2017). doi:10.1259/bjr.20160239.– main endpoint not consistent with our search strategy (DMFS)
7. Kim, Kyung Hwan, Jeongshim Lee, Jee Suk Chang, Chang Geol Lee, Mijin Yun, Eun Chang Choi, Se-Heon Kim, and Ki Chang Keum. “Prognostic Value of FDG-PET Volumetric Parameters in Patients with p16-Positive Oropharyngeal Squamous Cell Carcinoma Who Received Curative Resection Followed by Postoperative Radiotherapy or Chemoradiotherapy: PET Parameters in p16-Positive Oropharyngeal SCC.” Head & Neck 38, no. 10 (October 2016): 1515–24. doi:10.1002/hed.24465– whole cohort managed by surgery
8. Laskar, SarbaniGhosh, Gunjan Baijal, Venkatesh Rangarajan, Nilendu Purandare, Manju Sengar, Sneha Shah, Tejpal Gupta, et al. “Fluorodeoxyglucose-Positron Emission Tomography in Carcinoma Nasopharynx: Can We Predict Outcomes and Tailor Therapy Based on Postradiotherapy Fluorodeoxyglucose-Positron Emission Tomography?” Indian Journal of Medical and Paediatric Oncology 37, no. 1 (2016): 47. doi:10.4103/0971-5851.177030– lack of adequate information on FDG-PET semi-quantitative features
9. Lin, Y.-C., S.-W. Chen, T.-C. Hsieh, K.-Y. Yen, S.-N. Yang, Y.-C. Wang, and C.-H. Kao. “Risk Stratification of Metastatic Neck Nodes by CT and PET in Patients with Head and Neck Cancer Receiving Definitive Radiotherapy.” Journal of Nuclear Medicine 56, no. 2 (February 1, 2015): 183–89. doi:10.2967/jnumed.114.148023. –main endpoint not consistent with our search strategy (NRFS)
10. Mehanna, Hisham, Wai-Lup Wong, Christopher C. McConkey, Joy K. Rahman, Max Robinson, Andrew G. J. Hartley, Christopher Nutting, et al. “PET-CT Surveillance versus Neck Dissection in Advanced Head and Neck Cancer.” The New England Journal of Medicine 374, no. 15 (April 14, 2016): 1444–54. doi:10.1056/NEJMoa1514493. – lack of information on FDG-PET semi-quantitative features
11. Moeller, Benjamin J., Vishal Rana, Blake A. Cannon, Michelle D. Williams, Erich M. Sturgis, Lawrence E. Ginsberg, Homer A. Macapinlac, et al. “Prospective Risk-Adjusted [ ^18^ F]Fluorodeoxyglucose Positron Emission Tomography and Computed Tomography Assessment of Radiation Response in Head and Neck Cancer.” Journal of Clinical Oncology 27, no. 15 (May 20, 2009): 2509–15. doi:10.1200/JCO.2008.19.3300.– not validated endpoint for our search strategy (diagnostic accuracy)
12. Ng, Shu-Hang, Chien-Yu Lin, Sheng-Chieh Chan, Yu-Chun Lin, Tzu-Chen Yen, Chun-Ta Liao, Joseph Tung-Chieh Chang, et al. “Clinical Utility of Multimodality Imaging with Dynamic Contrast-Enhanced MRI, Diffusion-Weighted MRI, and 18F-FDG PET/CT for the Prediction of Neck Control in Oropharyngeal or Hypopharyngeal Squamous Cell Carcinoma Treated with Chemoradiation.” Edited by Pek-Lan Khong. PLoS ONE 9, no. 12 (December 22, 2014): e115933. doi:10.1371/journal.pone.0115933.– main investigation on imaging different than FDG (MR)
13. Paidpally, V., A. K. Tahari, S. Lam, K. Alluri, S. Marur, W. Koch, R. L. Wahl, and R. M. Subramaniam. “Addition of 18F-FDG PET/CT to Clinical Assessment Predicts Overall Survival in HNSCC: A Retrospective Analysis with Follow-up for 12 Years.” Journal of Nuclear Medicine 54, no. 12 (December 1, 2013): 2039–45. doi:10.2967/jnumed.113.121285. – not validated endpoint for our search strategy (diagnostic accuracy)
14. Vainshtein, Jeffrey M., Matthew E. Spector, Matthew H. Stenmark, Carol R. Bradford, Gregory T. Wolf, Francis P. Worden, Douglas B. Chepeha, et al. “Reliability of Post-Chemoradiotherapy F-18-FDG PET/CT for Prediction of Locoregional Failure in Human Papillomavirus-Associated Oropharyngeal Cancer.” Oral Oncology 50, no. 3 (March 2014): 234–39. doi:10.1016/j.oraloncology.2013.12.003- not validated endpoint for our search strategy (diagnostic accuracy)
15. Wong, K. H., R. Panek, L. Welsh, D. Mcquaid, A. Dunlop, A. Riddell, I. Murray, et al. “The Predictive Value of Early Assessment After 1 Cycle of Induction Chemotherapy with 18F-FDG PET/CT and Diffusion-Weighted MRI for Response to Radical Chemoradiotherapy in Head and Neck Squamous Cell Carcinoma.” Journal of Nuclear Medicine 57, no. 12 (December 1, 2016): 1843–50. doi:10.2967/jnumed.116.174433.– early time point of assessment (3 months)
16. Xiao, WeiWei, AnAn Xu, Fei Han, XiaoPing Lin, LiXia Lu, GuanZhu Shen, ShaoMin Huang, Wei Fan, XiaoWu Deng, and Chong Zhao. “Positron Emission Tomography–computed Tomography before Treatment Is Highly Prognostic of Distant Metastasis in Nasopharyngeal Carcinoma Patients after Intensity-Modulated Radiotherapy Treatment: A Prospective Study with Long-Term Follow-Up.” Oral Oncology 51, no. 4 (April 2015): 363–69. doi:10.1016/j.oraloncology.2015.01.009.– not validated endpoint for our search strategy (DMFS)
17. Yao, Min, Russell B. Smith, Henry T. Hoffman, Gerry F. Funk, Minggen Lu, Yusuf Menda, Michael M. Graham, and John M. Buatti. “Clinical Significance of Postradiotherapy [18F]-Fluorodeoxyglucose Positron Emission Tomography Imaging in Management of Head-and-Neck Cancer—A Long-Term Outcome Report.” International Journal of Radiation Oncology*Biology*Physics 74, no. 1 (May 2009): 9–14. doi:10.1016/j.ijrobp.2008.07.019.- lack of information on FDG-PET semi-quantitative features
