# Supplementary material for: What is the prognostic impact of FDG PET in locally advanced head and neck squamous cell carcinoma treated with concomitant chemo-radiotherapy? A systematic review and meta-analysis
Source: Eur J Nucl Med Mol Imaging. 2018 Jun 9;45(12):2122–38. doi: 10.1007/s00259-018-4065-5 (PMC6182396; doi:10.1007/s00259-018-4065-5)
Supplement: Supplementary file 3 — (DOCX 21 kb) [file 259_2018_4065_MOESM3_ESM.docx]

| **Authors [ref]** | **Year** | **Study design** | **Domain 1: Patient Selection** | | **Domain 2: Index Test** | | **Domain 3: Reference Standard** | | **Domain 4: Flow and Timing** |
| --- | --- | --- | --- | --- | --- | --- | --- | --- | --- |
|  |  |  | **Risk of bias** | **Concerns regarding applicability** | **Risk of bias** | **Concerns regarding applicability** | **Risk of bias** | **Concerns regarding applicability** | **Risk of bias** |
| Akagunduz OO [18] | 2015 | Retrospective | 3 | 1 | 1 | 1 | 1 | 1 | 1 |
| Castaldi P [19] | 2012 | Retrospective | 3 | 1 | 3 | 1 | 3 | 1 | 1 |
| Castelli J [20] | 2017 | Retrospective | 3 | 1 | 1 | 1 | 1 | 1 | 1 |
| Chen SW [21] | 2014 | Prospective | 1 | 1 | 3 | 1 | 3 | 1 | 1 |
| Cheng NM [22] | 2013 | Retrospective | 3 | 1 | 1 | 1 | 1 | 1 | 1 |
| Man KC [23] | 2009 | Retrospective | 3 | 1 | 1 | 1 | 1 | 1 | 1 |
| Higgins KA [24] | 2012 | Retrospective | 3 | 1 | 3 | 1 | 3 | 1 | 1 |
| Katahira-Suzuki R [25] | 2015 | Retrospective | 3 | 1 | 1 | 1 | 1 | 1 | 1 |
| Kim R [26] | 2016 | Retrospective | 3 | 1 | 1 | 1 | 1 | 1 | 1 |
| Koyasu S [27] | 2014 | Retrospective | 3 | 2 | 3 | 1 | 3 | 1 | 1 |
| Lin P [28] | 2017 | Retrospective | 3 | 1 | 3 | 1 | 3 | 1 | 2 |
| Marcus C [29] | 2014 | Retrospective | 3 | 1 | 3 | 1 | 3 | 1 | 1 |
| Matoba M [30] | 2017 | Prospective | 1 | 1 | 3 | 1 | 3 | 1 | 1 |
| Min M [31] | 2016 | Retrospective | 3 | 1 | 3 | 1 | 3 | 1 | 2 |
| Min M [32] | 2015 | Retrospective | 3 | 1 | 1 | 1 | 1 | 1 | 2 |
| Miyabe J [34] | 2017 | Retrospective | 3 | 1 | 1 | 1 | 1 | 1 | 1 |
| Murphy JD [35] | 2011 | Retrospective | 3 | 1 | 1 | 1 | 1 | 1 | 1 |
| Ng SH [36] | 2016 | Prospective | 1 | 1 | 1 | 1 | 1 | 1 | 1 |
| Park GC [37] | 2013 | Retrospective | 3 | 2 | 3 | 1 | 3 | 1 | 1 |
| Rasmussen JH [38] | 2015 | Retrospective | 3 | 1 | 3 | 1 | 3 | 1 | 1 |
| Romesser PB [39] | 2014 | Retrospective | 3 | 1 | 1 | 1 | 1 | 1 | 1 |
| Schwartz DL [40] | 2015 | Prospective | 2 | 1 | 2 | 1 | 2 | 1 | 2 |
| Van Den Wyngaer T [42] | 2016 | Prospective | 1 | 1 | 1 | 1 | 1 | 1 | 1 |
| Yoon YH [43] | 2014 | Retrospective | 3 | 1 | 1 | 1 | 1 | 1 | 1 |
| Zschaeck S [44] | 2017 | Retrospective | 3 | 2 | 1 | 1 | 1 | 1 | 2 |
